# Vacuum-assisted biopsy system for breast lesions: a potential therapeutic approach

**DOI:** 10.3389/fonc.2023.1230083

**Published:** 2023-08-01

**Authors:** Yue Zhu, Xingyan Chen, He Dou, Yuqi Liu, Fucheng Li, Youyu Wang, Min Xiao

**Affiliations:** Department of Breast Surgery, Harbin Medical University Cancer Hospital, Harbin, Heilongjiang, China

**Keywords:** vacuum-assisted breast biopsy system, mammotome minimally invasive surgery, breast ultrasonography, breast imaging-reporting and data system (BI-RADS), mammography, malignant lesions

## Abstract

**Purpose:**

The primary objective is to optimize the population eligible for Mammotome Minimally Invasive Surgery (MIS) by refining selection criteria. This involves maximizing procedure benefits, minimizing malignancy risk, and reducing the rate of malignant outcomes.

**Patients and methods:**

A total of 1158 female patients who came to our hospital from November 2016 to August 2021 for the Mammotome MIS were analyzed retrospectively. Following χ2 tests to screen for risk variables, binary logistic regression analysis was used to determine the independent predictors of malignant lesions. In addition, the correlation between age and lesion diameter was investigated for BI-RADS ultrasound (US) category 4a lesions in order to better understand the relationship between these variables.

**Results:**

The malignancy rates of BI-RADS US category 3, category 4a and category 4b patients who underwent the Mammotome MIS were 0.6% (9/1562), 6.4% (37/578) and 8.3% (2/24) respectively. Malignant lesions were more common in patients over the age of 40, have visible blood supply, and BI-RADS category 4 of mammography. In BI-RADS US category 4a lesions, the diameter of malignant tumor was highly correlated with age, and this correlation was strengthened in patients over the age of 40 and with BI-RADS category 4 of mammography.

**Conclusion:**

The results of this study demonstrate that the clinical data and imaging results, particularly age, blood supply, and mammography classification, offer valuable insights to optimize patients’ surgical options and decrease the incidence of malignant outcomes.

## Introduction

Currently, breast cancer is the primary cause of cancer-related death in women and has the greatest incidence of malignant tumors worldwide (1). For breast imaging examinations, it is generally recommended that women initiate early screening for breast cancer at the age of 40 (2). Early screening is a cost-effective and straightforward approach to assess asymptomatic women for breast cancer (3). In addition to clinical and imaging examinations, breast cancer knowledge promotion and breast self-examination are also important components of comprehensive breast cancer care. With the pervasive publicity of the concept of early screening and the continuous change of people’s lifestyle, the probability of women being detected with breast lesions has increased significantly. Therefore, the physical and mental health of patients who are diagnosed with breast lesions are extremely vulnerable to severe effects (4). Ultrasound is a non-radiation and non-invasive method commonly used for breast examination, but its primary limitation is its lower specificity and the potential for increased false positive results (5). The Breast Imaging-Reporting and Data System classifies breast tumors into six groups based on various ultrasound characteristics (BI-RADS US). Most BI-RADS US category 3 lesions are benign, while category 4 lesions suggest an increased risk of malignancy. For example, the malignant probability of category 4a is 2% to 10%, category 4b is 10% to 50%, and category 4c is 50% to 95% (6). The unnecessary invasive examination can be minimized by screening and observing category 3 and category 4a patients on a regular basis (7, 8). Surgical resection is typically advised if the patient’s daily life, physical health, or mental health are affected by these low-risk breast lesions (9).

In comparison to other treatment modalities, surgical intervention has consistently held a pivotal role as the fundamental component of comprehensive breast tumor management throughout its history (10). Traditional breast surgery has lengthy preparation, surgical trauma, blood loss, and visible scars. With research advancements and patient preferences, breast surgeons’ treatment approach has significantly evolved (11). Johnson & Johnson company of the United States launched the vacuum-assisted breast biopsy (VABB) system (The Mammotome System) in 1995, which was mainly used for the location and biopsy of suspicious breast lesions at the initial stage rather than treatment (12). However, the diagnostic advantage of Mammotome system is not particularly obvious, and few doctors only use it for breast tumor biopsy. Later, for small breast masses with negative palpation, clinicians removed the tumor completely while using this equipment for biopsy, so as to accomplish the goal of treatment, and obtained satisfactory results (13). Ultrasound-guided Mammotome Minimally Invasive Surgery (MIS) is a popular therapy option among patients due to the benefits of local anesthesia, tiny incisions, fewer scars, a low incidence of postoperative complications, and rapid wound healing (14, 15). The indications and contraindications of Mammotome MIS are more stringent than those of traditional surgery, but as instruments develop and experience accumulates, the scope of contraindications and indications for this procedure is changing slightly (16, 17).

Currently, the Mammotome system is extensively utilized for the removal of suspected benign breast tumors (18, 19). Breast biopsies are commonly performed using either 14-gauge core biopsy (CB) needles or the VABB technique. The VABB method employs larger gauge probes, ranging from 11 to 7-gauge, compared to CB (20). Unlike CB, the VABB technique offers the advantage of complete lesion removal while also providing histological verification (21). Typically, clinicians relied on chief complaints, medical history, and imaging reports to make an initial assessment of the patient’s condition. Patients who suspected their lesion to be benign were advised to consider MIS as an option. It not only fulfills the patients’ aesthetic concerns but also successfully achieves the therapeutic objective, thereby enhancing overall patient satisfaction (22, 23). This study conducted a comprehensive review and analysis of the clinical characteristics and imaging data of female patients with BI-RADS 3, 4a, and 4b lesions who underwent Mammotome MIS. The aim was to enhance the detection accuracy of malignant lesions and strengthen the intervention and treatment of benign lesions using Mammotome MIS. Due to the high malignancy rate (50%-95%) observed in category 4c lesions and the classification of category 5 for lesions with a malignancy rate exceeding 95%, MIS is generally not recommended as a preferred clinical option (24). In addition, given the increasing number of patients with BI-RADS 4a lesions opting for MIS and the relatively high malignancy rate associated with this category, our study also focused on investigating the relationship between age and lesion diameter, aiming to gain deeper insights into the correlation between these variables.

## Methods

### Patients

**Figure 1:**
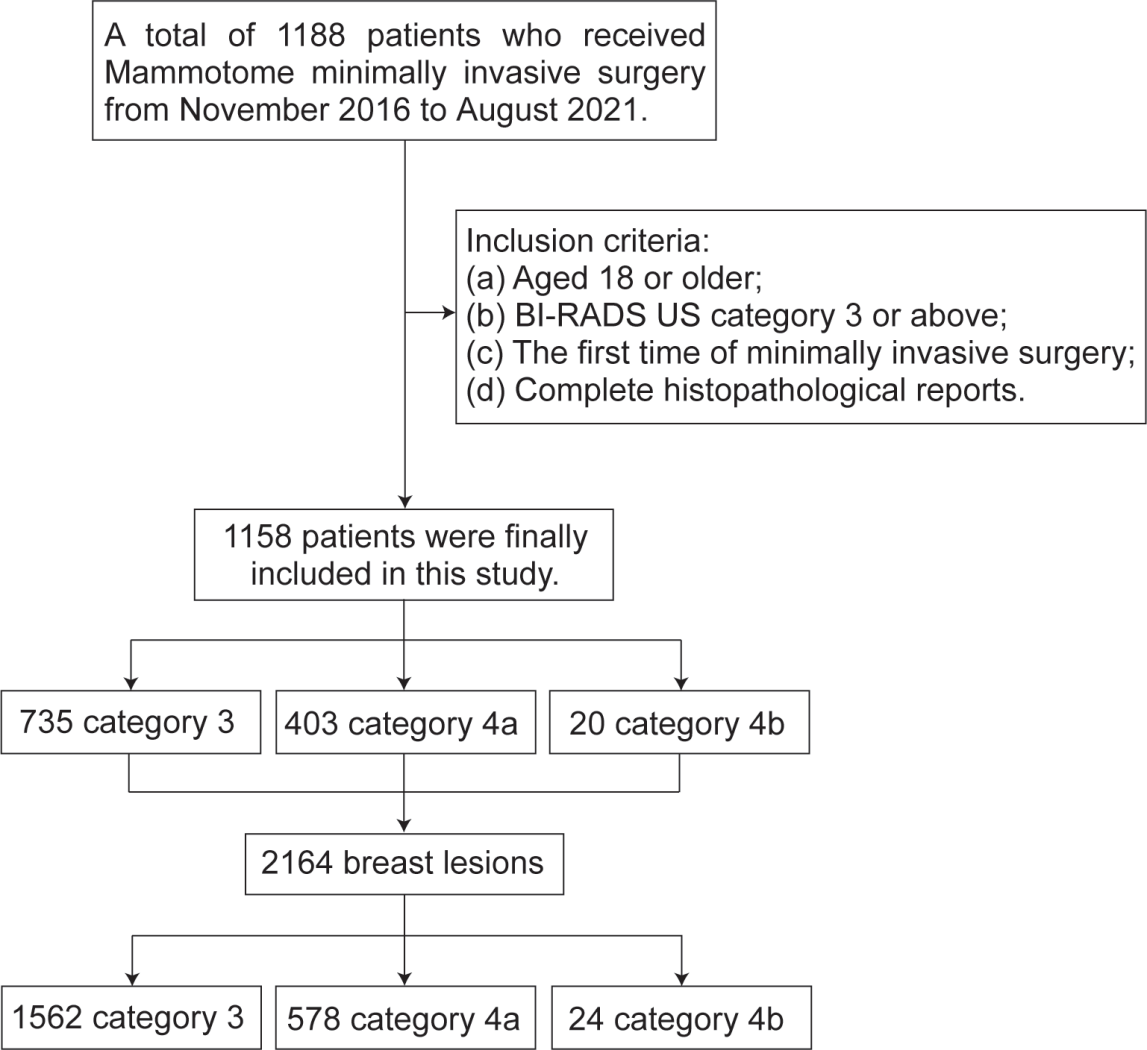
Flowchart of patient selection.

This retrospective study was approved by the Research Ethics Review Committee of the Harbin Medical University Cancer Hospital. Using the digital integrated management system of medical records from hospital, we collected 1188 female patients who underwent Mammotome MIS from November 2016 to August 2021. Screening based on the following inclusion criteria: (a) Aged 18 or older; (b) The results of conventional ultrasound examination were BI-RADS (BI-RADS US) category 3 or above; (c) Underwent Mammotome MIS for the first time; (d) Have a complete histopathological report. Finally, we decided to include 1158 patients with a total of 2164 breast lesions. The BI-RADS category of patient is defined according to the highest BI-RADS category of each patient’s lesion. According to the results of ultrasound diagnosis, the patients and the lesions were separately divided into three groups: BI-RADS US category 3 (may be benign), category 4a (low grade suspected malignant) and category 4b (moderately suspected malignant) (**Figure 1**).

### Variables

**Figure 2:**
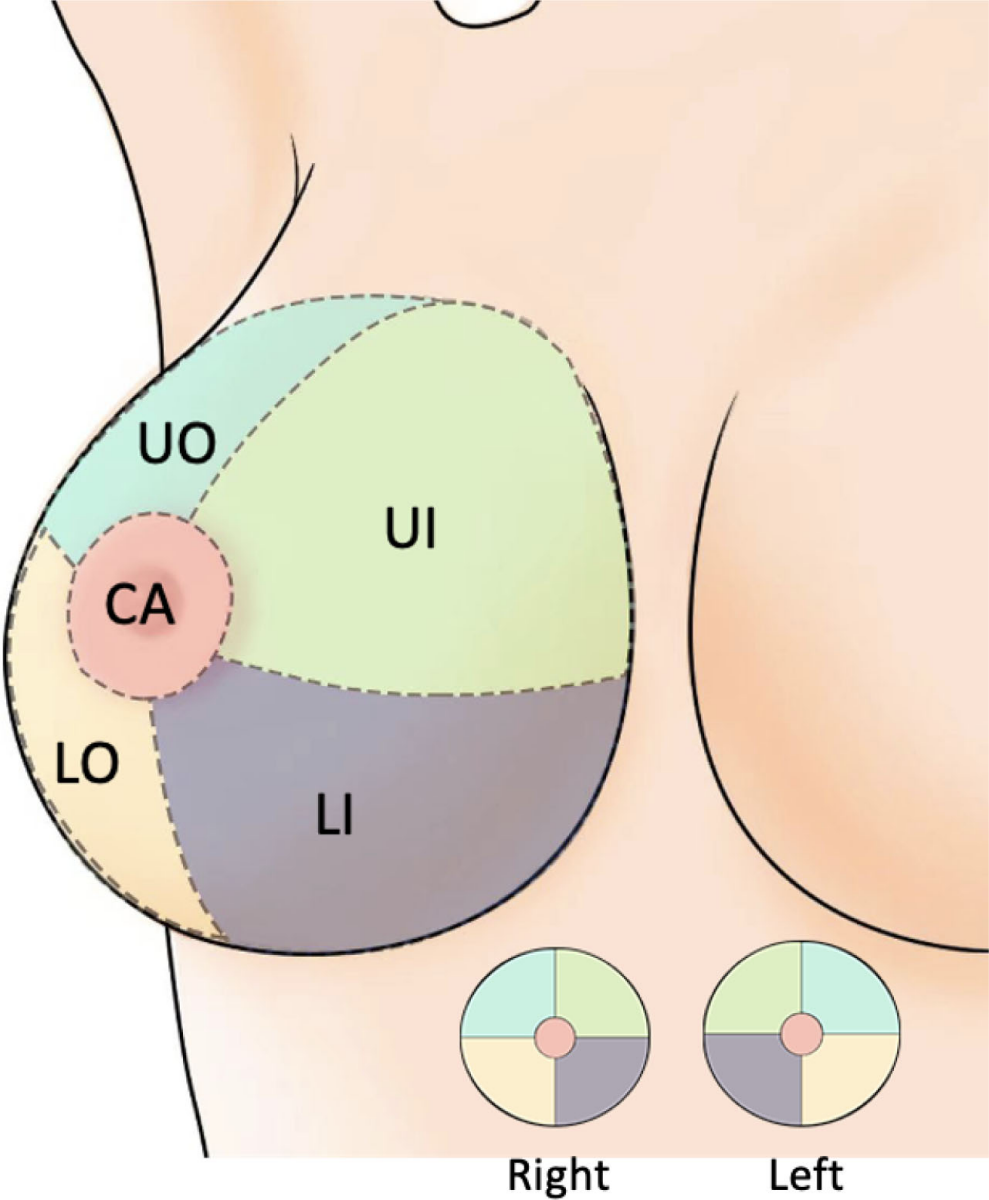
Breast quadrant division. UO, Upper Outer; LO, Lower Outer; LI, Lower Inner; UI, Upper Inner; CA, Central Area.

The basic clinical data included the patient’s age (< 40 years/≥ 40 years), menarche age (< 15 years/≥ 15 years), the location of the lesions (left/right) and quadrant position (UO/UI/LO/LI/CA) (**Figure 2**). The imaging results were jointly evaluated by both diagnostic doctors and audit doctors, including maximal lesion diameter (≥ 20 mm/< 20 mm), blood supply (invisible/visible), mammography BI-RADS category, calcification (invisible/single/multiple/unidentified). The ultrasound-guided biopsy device used in this study is Mammotome breast biopsy system (Tai Weikang Medical Devices (Shanghai) Co., Ltd.). The pathological results of all lesions were divided into benign and malignant. Malignant tumors included intraductal carcinoma in situ, invasive ductal carcinoma, solid papillary carcinoma and mucinous carcinoma. Patients with malignant diagnosis need to undergo a second operation, and the supplementary information includes the mode of operation, pathological results, histological grade, number of lymph nodes, immunohistochemical results, Ki-67 index value, accompanying with or without Intravascular tumor thrombus.

### Statistical analysis

R software (V4.2.2) is used for all statistical analysis. The quantitative variables (patient’s age, menarche age and maximal lesion diameter) were expressed as mean ± standard deviation. χ2 test or Fisher’s exact test is used to analyze qualitative variables. Binary logistic regression analysis was used to find the potentially independent risk factors of benign and malignant tumors in BI-RADS category 4a. P values less than 0.05 are considered statistically significant.

## Results

### Baseline characteristics of patients and their lesions

The basic clinical data of 1158 patients with a total of 2164 breast lesions are summarized in **Table 1**. The patients ranged in age from 18 to 72 years (mean, 38.4 years). Comparatively, it has been shown that patients in BI-RADS US category 3 tend to be younger than patients in category 4a and category 4b, and that this difference is statistically significant (3 vs 4a, P<0.001; 3 vs 4b, P=0.006). However, the menarche age was comparable and unrelated to the BI-RADS US category. Based on our findings, a significant number of patients presented multiple lesions, with 1562 (72.2%) categorized as category 3, 578 (26.7%) as category 4a, and 24 (1.1%) as category 4b. Unsurprisingly, the UO quadrant (47%) had the highest incidence rate of lesions, followed by the UI quadrant (21.8%) and the LO quadrant (20.8%). The CA quadrant had the lowest incidence rate of lesions (0.9%). Furthermore, a consistent pattern was observed across the different types of lesions categorized by BI-RADS. The diameter of the lesion was primarily less than 20mm (12.9 ± 6.0, 85.9%) due to Mammotome equipment limitations. The visible blood supply increased as the BI-RADS category increased, and there were statistical differences (3 vs 4a, P<0.001; 3 vs 4b, P<0.001; 4a vs 4b, P<0.001). In the mammography BI-RADS category, we discovered that the category 3 and lower are more prevalent (58.3%). BI-RADS US category 4a of lesions have a higher prevalence BI-RADS category 4 of mammography than BI-RADS US category 3 of lesions (P<0.001). Additionally, there were notable variations in calcification between the three types of BI-RADS (3 vs 4a, P<0.001; 3 vs 4b, P<0.001; 4a vs 4b, P=0.003), and category 4a had a considerably higher incidence of multiple calcifications (38.8%). Among the lesions analyzed, there were 2116 normal lesions and 48 malignant lesions. The malignancy rates varied across different BI-RADS US categories, with rates of 0.6% (9/1562) for category 3, 6.4% (37/578) for category 4a, and 8.3% (2/24) for category 4b.

**Table 1:** Clinical data of patients and lesions based on BI-RADS US category and comparison of difference between categories.

Category	Total	BI-RADS US category			P-Value			
		3	4a	4b	3 vs 4a	3 vs 4b	4a vs 4b	All
Number of patients	1158 (100%)	735 (63.5%)	403 (34.8%)	20 (1.7%)				
Age (years)					<0.001	0.006	0.169	<0.001
< 40	636 (54.9%)	446 (60.7%)	184 (45.7%)	6 (30.0%)				
≥ 40	522 (45.1%)	289 (39.3%)	219 (54.3%)	14 (70.0%)				
Mean ± SD	38.4 ± 9.8	36.8 ± 9.6	41.0 ± 9.8	43.9 ± 7.7				
Range (min-max)	(18-72)	(18-66)	(18-72)	(30-58)				
Menarche age (years)					0.574	0.587	0.486	0.712
< 15	768 (66.3%)	484 (65.9%)	272 (67.5%)	12 (60.0%)				
≥ 15	390 (33.7%)	251 (34.1%)	131 (32.5%)	8 (40.0%)				
Mean ± SD	14.2 ± 1.4	14.2 ± 1.4	14.1 ± 1.4	14.4 ± 1.4				
Range (min-max)	(11-20)	(11-19)	(11-20)	(13-19)				
Number of lesions	2164 (100%)	1562 (72.2%)	578 (26.7%)	24 (1.1%)				
Location					0.731	0.312	0.359	0.577
Left	1119 (51.7%)	813 (52.0%)	296 (51.2%)	10 (41.7%)				
Right	1045 (48.3%)	749 (48.0%)	282 (48.8%)	14 (58.3%)				
Quadrant					0.476	0.089	0.054	0.15
UO	1017 (47.0%)	739 (47.3%)	260 (45.0%)	18 (75%)				
LO	450 (20.8%)	324 (20.7%)	125 (21.6%)	1 (4.2%)				
LI	206 (9.5%)	152 (9.7%)	52 (9.0%)	2 (8.3%)				
UI	472 (21.8%)	336 (21.5%)	133 (23.0%)	3 (12.5%)				
CA	19 (0.9%)	11 (0.7%)	8 (1.4%)	0				
Maximal lesion diameter (mm)					0.092	0.409	0.193	0.154
< 20	1859 (85.9%)	1331 (85.2%)	509 (88.1%)	19 (79.2%)				
≥ 20	305 (14.1%)	231 (14.8%)	69 (11.9%)	5 (20.8%)				
Mean ± SD	12.9 ± 6.0	13.0 ± 6.0	12.5 ± 5.8	14.8 ± 6.7				
Range (min-max)	(2-43)	(2-43)	(3-38)	(6-29)				
Blood supply					<0.001	<0.001	<0.001	<0.001
Invisible	1738 (80.3%)	1310 (83.9%)	420 (72.7%)	8 (33.3%)				
Visible	426 (19.7%)	252 (16.1%)	158 (27.3%)	16 (66.7%)				
Mammography BI-RADS					<0.001	0.037	0.219	<0.001
0∼3	1262 (58.3%)	887 (56.8%)	356 (61.6%)	19 (79.2%)				
4	292 (13.5%)	161 (10.3%)	128 (22.1%)	3 (12.5%)				
Unidentified	610 (28.2%)	514 (32.9%)	94 (16.3%)	2 (8.3%)				
Calcification					<0.001	<0.001	0.003	<0.001
Invisible	741 (34.2%)	518 (33.2%)	216 (37.3%)	7 (29.2%)				
Single	156 (7.2%)	105 (6.7%)	44 (7.6%)	7 (29.2%)				
Multiple	657 (30.4%)	425 (27.2%)	224 (38.8%)	8 (33.3%)				
Unidentified	610 (28.2%)	514 (32.9%)	94 (16.3%)	2 (8.3%)				
Diagnose					<0.001	<0.001	0.706	<0.001
Benign	2116 (97.8%)	1553 (99.4%)	541 (93.6%)	22 (91.7%)				
Malignant	48 (2.2%)	9 (0.6%)	37 (6.4%)	2 (8.3%)				

BI-RADS, Breast imaging-reporting and data system; US, Ultrasound; UO, Upper Outer; LO, Lower Outer; LI, Lower Inner; UI, Upper Inner; CA, Central Area; Unidentified denotes the absence of a mammography imaging report for the patient.

### Pathological type of breast lesions

Among benign lesions, fibroadenoma (853/2116, 40.3%) was the most common, followed by adenosis (594/2116, 28.1%) and fibroadenoma with adenosis (552/2116, 26.1%). Notably, the numbers of intraductal papilloma in BI-RADS US category 3 and category 4a were similar, but the incidence was different (31/1553 vs 33/541, 2.0% vs 6.1%). In malignant lesions, the main pathological type was invasive ductal carcinoma (IDC) (27/48, 56.3%), followed by ductal carcinoma *in situ* (DCIS) (19/48, 39.6%). The number of malignant lesions diagnosed in category 4a was found to be the highest. In addition, two rare special type breast cancers were diagnosed, namely solid papillary carcinoma (SPC) and mucinous carcinoma (MC) (**Table 2**).

**Table 2:** Pathological types of benign and malignant lesions.

BI-RADS US category	3	4a	4b	Total
Benign lesions	1553	541	22	2116
Fibroadenoma	655	188	10	853
Adenosis	402	187	5	594
Fibroadenoma with adenosis	433	113	6	552
Intraductal papilloma	31	33	0	64
Benign lobular tumor	13	4	1	18
Benign breast tissue	9	6	0	15
Inflammation	3	3	0	6
Hamartoma	3	2	0	5
Atypical ductal hyperplasia	1	1	0	2
Breast Radial Scar	0	2	0	2
Cyst	1	1	0	2
Tubular adenoma	1	1	0	2
Borderline lobular tumor	1	0	0	1
Malignant lesions	9	37	2	48
Invasive ductal carcinoma	3	22	2	27
Ductal carcinoma in situ	5	14	0	19
Solid papillary carcinoma	1	0	0	1
Mucinous carcinoma	0	1	0	1

### Comparison of characteristics between benign and malignant lesions

By comparing the basic data and imaging features of patients with benign and malignant lesions, it was found that negative results included factors such as menarche age, lesion location, quadrant division, and diameter size. Compared with benign lesions, patients older than 40 years old had a significantly higher probability of malignant lesions (P<0.001). There was a significant increase in malignant lesions with visible blood supply (P<0.001), BI-RADS category 4 of mammography (P<0.001), and multiple calcifications (P<0.001) (**Table 3**). We analyzed univariate and multivariate binary logistic regression analysis for these four significant risk variables. The results showed that aged 40 or above, visible blood supply, and BI-RADS category 4 of mammography were significant risk factors for breast cancer (**Table 4**).

**Table 3:** Benign vs malignant in lesions.

Category	Benign	Malignant	χ2	P-Value
Age (years)			23.476	<0.001
< 40	1185 (99.2%)	10 (0.8%)		
≥ 40	931 (96.1%)	38 (3.9%)		
Menarche age (years)			0.043	0.835
< 15	1397 (97.8%)	31 (2.2%)		
≥ 15	719 (97.7%)	17 (2.3%)		
Location			0.119	0.73
Left	1093 (97.7%)	26 (2.3%)		
Right	1023 (97.9%)	22 (2.1%)		
Quadrant			5.107	0.276
UO	988 (97.1%)	29 (2.9%)		
LO	445 (98.9%)	5 (1.1%)		
LI	201 (97.6%)	5 (2.4%)		
UI	463 (98.1%)	9 (1.9%)		
CA	19 (100%)	0		
Maximal lesion diameter (mm)			0.879	0.349
< 20	1820 (97.9%)	39 (2.1%)		
≥ 20	296 (97.0%)	9 (3.0%)		
Blood supply			12.292	<0.001
Invisible	1709 (98.3%)	29 (1.7%)		
Visible	407 (95.5%)	19 (4.5%)		
Mammography BI-RADS			103.653	<0.001
0∼3	1245 (98.7%)	17 (1.3%)		
4	262 (89.7%)	30 (10.3%)		
Unidentified	609 (99.8%)	1 (0.2%)		
Calcification			23.534	<0.001
Invisible	726 (98.0%)	15 (2.0%)		
Single	151 (96.8%)	5 (3.2%)		
Multiple	630 (95.9%)	27 (4.1%)		
Unidentified	609 (99.8%)	1 (0.2%)		

**Table 4:** Logistic regression analysis of the characteristics of benign and malignant lesions.

Category	Univariate		Multivariate	
	OR (95% CI)	P-Value	OR (95% CI)	P-Value
Age (years)	3.528 (1.653, 8.718)	0.003	3.008 (1.368, 7.591)	0.011
< 40				
≥ 40				
Blood supply	2.623 (1.342, 4.942)	0.003	2.28 (1.124, 4.478)	0.019
Invisible				
Visible				
Mammography BI-RADS	10.116 (5.291, 20.389)	<0.001	8.512 (4.397, 17.326)	<0.001
0∼3				
4				
Calcification	2.074 (1.108, 4.029)	0.026	1.829 (0.950, 3.648)	0.077
Invisible				
Multiple				

OR, Odds ratio; 95% CI, 95% confidence interval.

### Correlation analysis of BI-RADS US category 4a lesions

**Figure 3:**
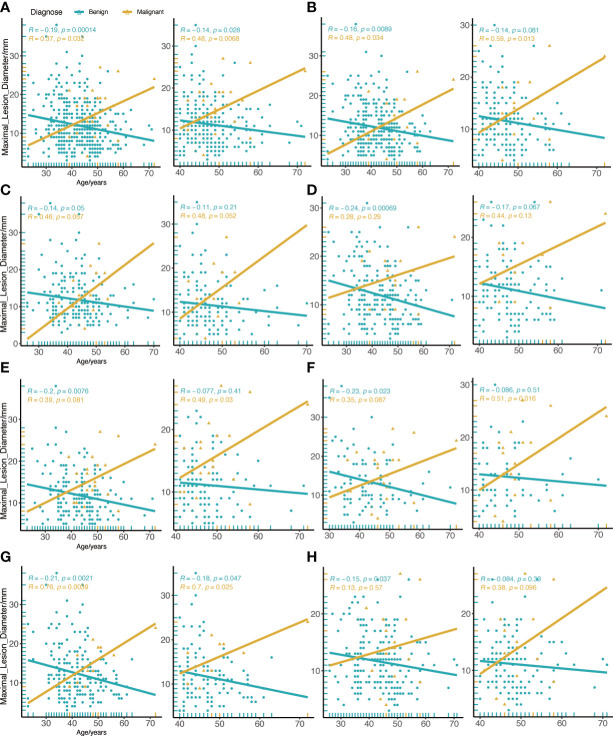
Correlation between age and maximal diameter of the lesion in all BI-RADS US category 4a. **(A)** All BI-RADS US category 4a patients. **(B)** Menarche age less than 15 years old. **(C)** Left breast lesions. **(D)** Right breast lesions. **(E)** Lesions in the UO quadrant. **(F)** the BI-RADS category 4 of mammography lesions. **(G)** Invisible calcification lesions. **(H)** Multiple calcification lesions. The left figure shows patients of all ages, and the right figure shows patients aged 40 or older.

We compared the differences between patients of all ages and patients over the age of 40. In general, we discovered that the diameter of a malignant tumor was positively correlated with age (R=0.37, P=0.032), whereas benign lesions were the inverse (R=-0.19, P<0.001). However, this association was stronger in cancer patients over the age of 40 (R=0.48, P=0.0068) (**Figure 3A**). The association between malignant tumor and diameter of patients older than 40 years old was further reinforced when the menarche age was less than 15 years old (R=0.59, P=0.013) (**Figure 3B**). According to the analysis of left and right breast lesions, it was found that the correlation between malignant tumor diameter and age of left breast was slightly more obvious than that of right breast, but there was no statistical difference (**Figures 3C, D**). In contrast to BI-RADS 4a patients in all age groups, we discovered that there was a strong correlation between the malignant tumor diameter and age in patients older than 40 years old in all lesions in the UO quadrant (R=0.49, P=0.03) (**Figure 3E**). Similar phenomena were also found in the BI-RADS category 4 of mammography lesions (R=0.51, P=0.016) (**Figure 3F**). In patients without calcification, there was a substantial correlation between the diameter of the malignant tumor and age (R=0.76, P=0.0039) (**Figure 3G**), but this phenomenon was not seen in patients with numerous calcifications (**Figure 3H**).

### Secondary supplementary surgery for malignant lesions

**Figure 4:**
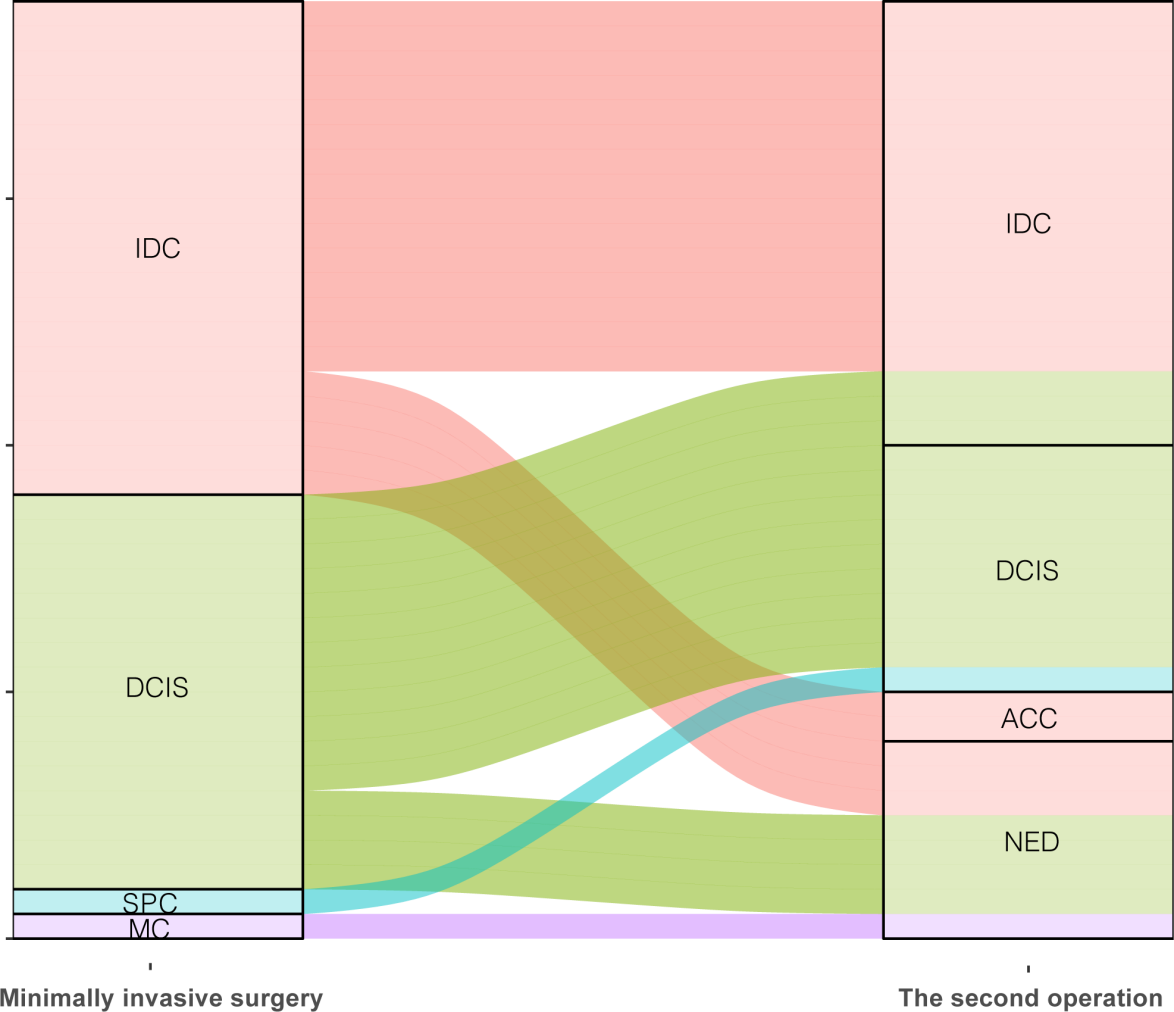
Comparison of pathological diagnosis results of two operations. IDC, Invasive ductal carcinoma; DCIS, Ductal carcinoma in situ; SPC, Solid papillary carcinoma; MC, Mucinous carcinoma; ACC, Adenoid cystic carcinoma; NED, No evidence of disease.

Among these patients with malignant lesions, 31 of them underwent secondary supplementary surgery at our hospital, and a total of 38 tumors were identified. **Table 5** shows the outcomes of secondary surgery for these malignant lesions. We observed that the second surgery had the highest proportion of breast-conserving surgery and sentinel lymph node biopsy (BCS+SLNB) (21/38, 55.3%). It was found that the pathological outcomes of the first Mammotome MIS and the second supplementary surgery were not exactly consistent. Among the IDC diagnosed by Mammotome MIS, the second pathological results were IDC, adenoid cystic carcinoma (ACC) and no evidence of disease (NED). Among the cases of DCIS, three cases were found to have transformed into IDC, while four cases showed NED. Furthermore, SPC transformed into DCIS, while MC showed NED (**Figure 4**). Based on the immunohistochemical findings, the vast majority of molecular subtypes were identified as Luminal A (33/38, 86.8%). Interestingly, low expression of Ki-67 was observed in 65.8% (25/38) of these malignant lesions.

**Table 5:** Pathological results of secondary supplementary surgery for malignant lesions.

Category	Total (n=38)	IDC (n=20)	DCIS (n=16)	SPC (n=1)	MC (n=1)
Second operation					
BCS+SLNB	21	6	14	1	0
M+SLNB	15	12	2	0	1
M+SLNB+ALND	1	1	0	0	0
MRM	1	1	0	0	0
Pathological types					
IDC	18	15	3	0	0
DCIS	10	0	9	1	0
ACC	2	2	0	0	0
NED	8	3	4	0	1
Grade					
I	3	3	0	0	0
II	11	9	2	0	0
III	4	3	1	0	0
None	20	5	13	1	1
Positive lymph nodes					
No	37	19	16	1	1
Yes	1	1	0	0	0
IHC					
Luminal A	33	18	13	1	1
Luminal B	1	1	0	0	0
TNBC	4	1	3	0	0
Ki-67					
≤10%	25	13	11	1	0
>10% and ≤25%	6	2	3	0	1
>25%	7	5	2	0	0
ITT					
No	37	19	16	1	1
Yes	1	1	0	0	0

BCS, Breast-conserving surgery; M, Mastectomy; MRM, Modified radical mastectomy; SLNB, Sentinel lymph node biopsy; ALND, Axillary lymph node dissection; IDC, Invasive ductal carcinoma; DCIS, Ductal carcinoma in situ; SPC, Solid papillary carcinoma; MC, Mucinous carcinoma; ACC, Adenoid cystic carcinoma; NED, No evidence of disease; IHC, Immunohistochemistry; ITT, Intravascular tumor thrombus.

## Discussion

From traditional surgery to MIS, breast tumor surgery has been committed to the development of “reduction” and “precision”. At the end of the 20th century, the theory of MIS progressively matured and endoscopic surgery developed rapidly (25). While it is true that MIS represents a relatively small portion of breast surgery procedures, it offers distinct advantages such as enhanced accuracy and minimal trauma (26). The maturity of these minimally invasive techniques and the success of clinical practice provide useful theories and techniques for the development of breast surgery. Ultrasound-guided Mammotome vacuum-assisted minimally invasive breast surgery, unlike laparoscopic surgery, does not involve the use of an endoscope. Currently, the indications for Mammotome MIS have been gradually expanded, allowing its application to the biopsy of various breast lesions. Under the guidance of imaging results and clinical experience, it may also be used to remove suspected benign lesions in addition to tissue biopsies.

In this study, we compare in detail the clinical basic data and imaging features of 1158 patients and 2164 lesions who underwent the Mammotome MIS. However, we discover that these patients still had a malignancy incidence of approximately 2.2%, with BI-RADS US category 4a lesions accounting for the highest proportion. Malignant lesions, as opposed to benign lesions, are more common in patients over the age of 40 and have visible blood supply, as well as BI-RADS category 4 of mammography. Usually, we recommend breast reexamination every six months for patients with BI-RADS US category 3 lesions (27). However, for patients with a psychological burden and a desire to maintain good breast appearance, we may recommend Mammotome MIS if the imaging findings show no obvious malignant features and the patients are willing to undergo lesion removal. The primary reason is that these patients have a very low malignancy rate, with the majority of them having carcinoma in situ. In the largest research to date, Berg et al. observe that the malignancy rate of BI-RADS US category 3 was 1.86% (28). This result is not in conflict with our data, as patients who have undergone Mammotome MIS have been preliminary screened. In a study to evaluate the accuracy of classification of category 3 lesions, computer-aided system also could reduce the misdiagnosis rate of malignant tumors (29).

The ultrasonic BI-RADS classification revised by the American College of Radiology (ACR) in 2013 defines category 4 lesions in more detail, and divides them into 4a, 4b and 4c (30). It has been argued that the subdivision of category 4 breast masses will not improve management, as all suspicious lesions still need to be clearly biopsied (31). In general, category 4a lesions require close observation for any changes, and biopsy should be performed when necessary to clarify the pathological nature. For category 4b lesions, patients are typically recommended to undergo biopsy in order to determine whether the lesion is benign or malignant. Our data reveals that patients with category 4b lesions represent a smaller subset in terms of the number of individuals who opted for Mammotome MIS, as compared to the other two categories. This is because category 4b lesions themselves have a 10% to 50% chance of developing malignancy, and our statistics also demonstrate a high incidence of malignancy. Therefore, most patients are advised to undergo core-needle biopsy rather than opting for Mammotome MIS.

Our data reveal that 6.4% of malignant events occurred in BI-RADS US category 4a patients who underwent Mammotome MIS, despite their clinical screening. For category 4a lesions, there is still an urgent need to explore other potential high-risk factors to reduce the malignant possibility of patients underwent Mammotome MIS. So far, various studies have tried to overcome the limitations of breast ultrasound screening, including the application of risk assessment models (32) and the development of auxiliary systems (33). There are a variety of breast cancer risk assessment models, and Gail model is one of the most widely used standard breast cancer risk assessment methods (34). In addition, for category 4a lesion, artificial intelligence also shows high diagnostic efficiency (35). Our research reveals that the majority of patients with category 4a malignant lesions are over 40 years old, which matches the data collection included in a predictive model (36), and that age is positively correlated with tumor diameter. Menarche age is not a major risk factor for distinguishing benign from malignant lesions, but in patients with menarche age less than 15 years old, the relationship between age and tumor diameter is further strengthened. Simultaneously, identical findings are made in patients with malignant lesions that lacked calcification.

When considering the use of vacuum biopsy as a therapeutic approach in cancer, it is important to exercise caution and carefully evaluate its applicability. International guidelines recommend the use of vacuum-assisted excision for breast lesions with a maximum diameter of 25 mm (37). This technique is considered appropriate and effective for the removal of such lesions. In some cases of resected tumors with larger diameters, the presence of peri-interventional inflammation around the biopsy area can pose challenges to tumor resection. This inflammation can complicate the surgical procedure and potentially impact the effectiveness of the treatment. Antonio et al. discovered that post-biopsy peripheral inflammation can transmit growth signals to remaining cancer cells or precancerous cells, leading to a negative impact on disease progression (38). This inflammation can complicate the surgical procedure and potentially impact the effectiveness of the treatment. In order to fully ensure the negative histopathology of the incision margin during the resection of the lesion, a second supplementary operation is required. This is due to the limitations of the Mammotome MIS. Therefore, MIS should be cautiously chosen for patients with clearly malignant lesions in order to prevent needless medical disputes.

## Conclusions

Patients in BI-RADS US category 3 who underwent Mammotome MIS tend to be younger compared to those in category 4a and 4b. Age, visible blood supply, and BI-RADS category 4 of mammography are potentially independent risk factors for breast malignancy. Additionally, we discovered a positive correlation between diameter size and age in malignant lesions by conducting a correlation analysis on BI-RADS US category 4a lesions. In patients with malignant lesions who are older than 40 years old, have an age of menarche younger than 15 years old, a mammography report greater than BI-RADS 4, and without calcifications, this positive correlation trend will further increase. High-risk groups with these factors should monitor lesion changes, undergo biopsy if need for pathology confirmation, and avoid using the vacuum biopsy system for treatment.

## Data availability statement

The original contributions presented in the study are included in the article/supplementary material. Further inquiries can be directed to the corresponding author.

## Ethics statement

The studies involving human participants were reviewed and approved by the institute research ethics committee of Harbin Medical University Cancer Hospital. The patients/participants provided their written informed consent to participate in this study.

## Author contributions

We confirm that the manuscript is original. MX put forward the idea and supervised this project. YZ was responsible for analyzing data and writing manuscript. The authors read and approved the final manuscript.
